# Handgrip Strength Features in Rheumatoid Arthritis Patients Assessed Using an Innovative Cylindrical-Shaped Device: Relationships With Demographic, Anthropometric and Clinical Variables

**DOI:** 10.1007/s10916-021-01778-9

**Published:** 2021-10-09

**Authors:** Fausto Salaffi, Marina Carotti, Sonia Farah, Luca Ceccarelli, Marco Di Carlo

**Affiliations:** 1 Rheumatology Clinic, Ospedale “Carlo Urbani”, Università Politecnica delle Marche, Jesi (Ancona), Italy; 2 Department of Radiology, Ospedali Riuniti, Università Politecnica delle Marche, Ancona, Italy; 3 U.O. di Radiologia – Ospedale degli Infermi, Azienda AUSL della Romagna, Faenza, Italy

**Keywords:** Rheumatoid arthritis, Handgrip strength, Cylindrical-shaped dynamometer, Disease activity indices, Disability

## Abstract

To investigate the relationship between handgrip strength (HGs) features, evaluated with an innovative cylindrical-shaped grip device, and demographic, anthropometric and clinical variables, in patients with rheumatoid arthritis (RA). Consecutive RA patients were prospectively enrolled for this cross-sectional study. For each patient were collected demographic, anthropometric, clinical data related to disease activity. HGs was assessed in terms of area under the force–time curve (AUC-FeT), peak grip force and time to reach the curve plateau. The correlations between the variables were studied with the Spearman’s rho correlation coefficient. The receiver operating characteristic (ROC) curve analysis was used to test the discriminant accuracy of HGs features in identifying patients in moderate/high disease activity. A multivariate analysis was performed to estimate the contribution of covariates on the AUC-FeT. A significant correlation was found among AUC-FeT, age, Simplified Disease Activity Index (SDAI), Ultrasound-Clinical Arthritis Activity (US-CLARA) (all at *p* < 0.0001), and body mass index (BMI) (*p* = 0.0001). Any correlation was found between HGs and radiographic damage. The discriminatory power of AUC-FeT was good [area under-ROC curve = 0.810 (95% CI 0.746–0.864)]. Variables significantly associated with AUC-FeT in multivariate analysis were age (*p* = 0.0006), BMI (*p* = 0.012), gender (*p* = 0.004), SDAI (*p* = 0.047) and US-CLARA (*p* = 0.023). HGs is negatively influenced by demographic (gender and age), anthropometric (BMI), and disease activity variables (SDAI and US-CLARA). These findings highlight the role of HGs in RA patients' functional impairment and disability.

## Introduction

Rheumatoid arthritis (RA) is a chronic inflammatory disease that affects people all over the world, with a 0.5% of prevalence in Italy [1]. RA affects a number of joints, with a large percentage of patients experiencing symptoms in hands and wrists. Joint pain and swelling are common symptoms, resulting in diminished hand strength and movement impairment [2]. Although there is some evidence that measures of disease activity may not fully reflect the regional impact of RA on the hands, indices such as the disease activity score with 28 joint count (DAS28) are the cornerstore of the assessment RA patients [3]. It might be argued that evaluating hand function should be done separately from a RA patient's disease activity evaluation [4]. Handgrip strength (HGs) has been shown to correlate with both objective and patient-reported outcome measures (PROMs) of disease activity and function [5, 6]. HGs can be used to assess a patient's ability to return to routine activities and job, as well as to track progress and compare the efficacy of different treatment strategies. Results coming from the VErA (Very Early Rheumatoid Arthritis) project has revealed that lower HGs in RA patients are related with a larger economic burden [7].

Several studies support the use of ultrasound (US) to detect and monitor joint inflammation and bone deterioration in individuals with RA. Although the joint count remains the most specific marker for RA assessment, several studies have recently been published that support the use of US to detect and monitor joint inflammation and bone deterioration in individuals with RA [8]. Current European Leaugue Againts Rheumatims (EULAR) recommendations on the use of imaging modalities in RA recognize the high sensitivity of US to detect joint pathologies, suggesting its extensive use in the assessment of disease activity [9]. Power Doppler US (PDUS) has enhanced its sensitivity in identifying low velocity flow at the microvascular level, making it a helpful method for assessing the degree of inflammation and disease activity in RA [10, 11].

Multimodal disease activity indices combining clinical and ultrasonographic data seem to have better reliability than clinical assessment alone [12, 13]. An Italian study conducted on a cohort of RA patients starting treatment with abatacept, demonstrated good metrological properties (validity, responsiveness and interpretability) compared to traditional disease activity indices, of a new composite index called Ultrasound-Clinical Arthritis Activity (US-CLARA) [14]. To date, there are no studies that have compared HGs with multimodal disease activity indices of in RA.

At present, it is also unknown whether or not hands radiological damage is associated with a reduction in HGs. With currently available tools such as the Simple Erosion Narrowing Score (SENS) [15], a simplified instrument based on the Sharp-van der Heijde Score (SHS), the assessment of radiographic damage is also accessible in daily clinical practice.

The evaluation of HGs can be performed with different types of dynamometer. To the best of our knowledge, no study to date has assessed HGs in RA using a cylindrical-shaped dynamometer.

Starting from these assumptions, the objective of this study was to investigate the relationship between HGs features and clinical variables, composite and multimodal disease activity indices, and radiographic damage.

## Methods

### Design and study population

In this observational cross-sectional study, consecutive RA patients were prospectively enrolled from January 2019 to December 2020. Patients were enrolled in the outpatients clinic of the Rheumatology Clinic of the Università Politecnica delle Marche, “Carlo Urbani” Hospital, Jesi (Ancona), Italy. The inclusion criteria were: age between 18 and 80 years, diagnosis of RA made according to the 2010 American College of Rheumatology (ACR)/EULAR criteria [16]. Exclusion criteria were represented by the presence of: limitations (such as visual impairments, lack of command of the Italian language) determining the impossibility to complete the clinimetric assessment, concurrent life-threatening disorders (such as active neoplasms, heart failure, severe chronic obstructive pulmonary disease, multiple sclerosis, extracorporeal dialysis), coexisting inflammatory crystal arthropaties (such as gout or calcium pyrophosphate deposition disease) of coexisting conditions capable of determining a reduction of HGs or altering the clinimetric assessment such as fibromyalgia.

### Assessment of demographic, anthropometric variables and comorbidities

Age, sex, disease duration (defined as time since diagnosis), level of education (primary, secondary, and university), body mass index (BMI), concomitant medication with glucocorticoids and traditional conventional syntethic disease modifying anti-rheumatic drugs (csDMARDs) or biological DMARDs (bDMARDs), and comorbidities were all included in the study. The comorbidities burden was estimated using the modified Rheumatic Disease Comorbidity Index (mRDCI) [17, 18], computed with the formula: 1* lung disease and [2* (myocardial infarction, other cardiovascular illnesses, or stroke) or 1* hypertension] and 1* (ulcer or other gastrointestinal disorders) and 2* kidney disease and 1* if BMI is > 30 kg/m^2^or 2* if BMI is > 35 kg/m^2^, and 1 for each of diabetes, fracture, depression, and cancer. The final mRDCI score ranges from 0 to 12 [17].

### Laboratory investigations

Standard laboratory methods were used to assess the erythrocyte sedimentation rate (ESR) (normal values 15 mm/1st hour in men and 20 mm/1st hour in women), the C-reactive protein (CRP) (expressed in mg/dl), the IgM-rheumatoid factor (RF) (measured by nephelometric method, Image Beckman), and the anti–citrullinated protein antibodies (ACPA) (measured by ImmunoFluoroMetric Assay, EliA CCP, ImmunoCAP 250, Phadia S.r.l., Italy).

### Assessment of HGs

HGs was measured using a cylindrical-shaped grip device with five force sensors (FRS-402, manufacted by Interlink Electronics) attached to a microcontroller (Arduino Mega 2560). This dynamometer represents a prototype and is not yet available on a large scale.

The grip force was measured by the grip device every 5 s for 30 s. This assessment allowed to draw the force–time (FeT) curves. The FeT curves are a graphical representation of muscular contraction force over time. The principal outcome for the purposes of this study was the estimation of the area under the FeT curve (AUC-FeT), with peak grip force and time to reach the curve plateau as secondary outcomes.

The HGs features were measured twice in the dominant with an interval of 5 min for fatigue recovery between the two measurements. The mean of the two values of the three parameters was used for the analyses [19]. HGs was assessed according to the American Society of Hand Therapists' recommendations for subject positioning [20]: patients in seated position, the shoulder adducted and neutrally rotated, the wrist was slightly extended, the elbow was flexed to roughly 90 degrees, and the forearm was in neutral position. Instructions were presented in a consistent manner to all patients.

### Disease activity indices

The Simplified Disease Activity Index (SDAI) and the US-CLARA were respectively used as composite and multimodal disease activity indices.

The SDAI is the algebraic sum of five unweighted, untransformed variables: 28-joint swollen and tender joints counts (SJC and TJC, respectively), physician and patient disease activity assessment (PhGA and PaGA, respectively) on 11-point numerical rating scales (NRS), and CRP (in mg/dl). The SDAI range is 0–86. Remission (REM), low disease activity (LDA), and moderate disease activity (MDA) has the predefined cut-off values of 3.3, 11 and 26, respectively [21].

The US-CLARA is a composite index that combines the Recent Onset Arthritis Disability (ROAD) questionnaire, the self-administered TJC, and the US evaluation into a single measure of disease activity [14, 22, 23]. The US examinations were carried out with a MyLab Class C US system (Esaote S.p.A., Genoa, Italy), equipped with a 6–18 MHz linear probe. A full description of the US-CLARA is provided in the validation study [14]. The final score of US-CLARA ranges from 0 to 10, and the following interpretability cut-off values have been proposed: REM < 2.0; 2.0 ≤ LDA < 3.0; 3 ≤ MDA < 4.8; and high disease activity (HDA) > 4.8.

### Radiographic assessment

Hands, wrists, and foot radiography were performed on all of the patients. The images were evaluated using the SENS by two experienced readers (FS and MC). The SENS scoring system evaluates the same joints as the SHS scoring technique. While SHS uses a 0–4 semiquantitative scale for joint space narrowing (JSN) and a 0–5 scale for erosions, SENS simply determines if an erosion is present (score 1) and whether JSN is present (score 1). The SENS has a maximum possible score of 86 [15].

### Statistical analysis

The MedCalc Statistical Software, version 19.0 (Ostend, Belgium), for Windows XP was used to analyze the data. For categorical variables, general descriptive statistics were described using numbers and percentages, while continuous variables were described using mean with standard deviation (SD), and median with interquartile range (IQR). The normal distribution was tested with the Kolmogorov–Smirnov test. Where appropriate, the percent differences between the groups were examined using a chi-square or Fisher's exact test. The Mann–Whitney U test was used to compare continuous variables among categories of grouped data.

The Spearman's rho was used to measure the degree of correlation between the variables.

Patients were categorized for disease severity states according to SDAI. The AUC-FeT differences according the categories were tested with the one-way analysis of variance (ANOVA).

Then, a receiver operating characteristic (ROC) curve analysis was used to test the discriminant accuracy of HGs measurements using SDAI (patients were categorized in MDA + HDA vs REM + LDA) as external criterion. AUC-ROC values between 0.7 and 0.8 suggest a fair discrimination, whereas values above 0.8 suggest a good discrimination. We used non-parametric resampling and the bias-corrected and accelerated approach to construct 95% confidence intervals (CIs) on 1000 bootstrapped samples. The non-parametric Wilcoxon signed ranks test was used to calculate and compare the AUC-ROCs.

Finally, the relative effect of individual variables (including among covariates age, sex, education, disease duration, BMI, mRDCI, SDAI, US-CLARA and SENS) on the AUC-FeT (considered as dependent variable) was assessed using a multivariate regression model approach. For all analyses, the level of significance was fixed at *p* < 0.05.

## Results

The final analysis included 186 RA patients (68.8% female), with a mean (SD) age of 55.88 (14.36) years, and a mean (SD) disease duration of 5.31 (4.18) years. The 95.1% was right-hand dominating. At least one csDMARD or bDMARD was taken by all the patients. Fifty-five (29.5%) patients were receveing a bDMARD, respectively 15 (27.3%) adalimumab, 14 (25.4%) etanercept, 11 (20.0%) golimumab, 9 (16.4%) abatacept, 4 (7.2%) tocilizumab, and 2 (3.6%) infliximab. Thirty-two patients (17.2%) were on oral corticosteroids, with a mean prednisone or equivalent dose of 4.9 mg/day (range 2.5–20), while 113 (60.8%) were given no-steroidal anti-inflammatory drugs on demand.

The mean values of the AUC-FeT, peak grip force, and time to reach the plateau of the curve were respectively 426.15 (150.90) Kg/30 s, 17.51 (6.03) Kg, and 13.17 (6.32) s. Males patients have a higher AUC-FeT than females [472.46 (131.89) Kg/30 s vs 406.63 (154.90) Kg/30 s; *p* = 0.014). Table 1 summarizes the clinical and demographic variables of the study cohort.

**Table 1:** Demographic and clinical characteristics of the patients

	Mean	Median	SD	IQR
Age (years)	55.81	55.00	14.36	44.00–68.00
Disease duration (years)	5.31	4.00	4.18	2.00–8.00
Educational level (years)	11.66	13.00	3.91	8.00–13.00
BMI (Kg/m^2^)	26.96	26.60	2.55	25.50–27.90
mRDCI (score 0–12)	1.90	2.00	1.67	0.00–3.00
SDAI (score 0–86)	22.02	18.40	14.86	10.20–33.44
US-CLARA (score 0–10)	4.34	4.16	2.52	2.33–6.18
SENS (score 0–86)	19.92	14.00	14.79	10.00–23.00
Handgrip strength AUC-FeT (Kg/30 s)	426.15	418.08	150.90	303.25–515.36
Handgrip strength peak grip force (Kg)	17.51	17.32	6.03	12.52–21.67
Time to reach maximum plateau of the curve (s)	13.17	12.50	6.32	7.50–17.50
RF positivity, n (%)	141 (75.80)			
ACPA positivity, n (%)	120 (64.51)			

*SD* standard deviation, *IQR* interquartile range, *BMI* body mass index, *mRDCI* modified Rheumatic Disease Comorbidity Index, *SDAI* Simplified Disease Activity Index, *US-CLARA* Ultrasound-Clinical Arthritis Activity, *SENS* Simple Erosion Narrowing Score, *AUC-FeT* area under the force–time curve, *RF* Rheumatoid Factor, *ACPA* anti–citrullinated protein antibodies

**Fig. 1:**
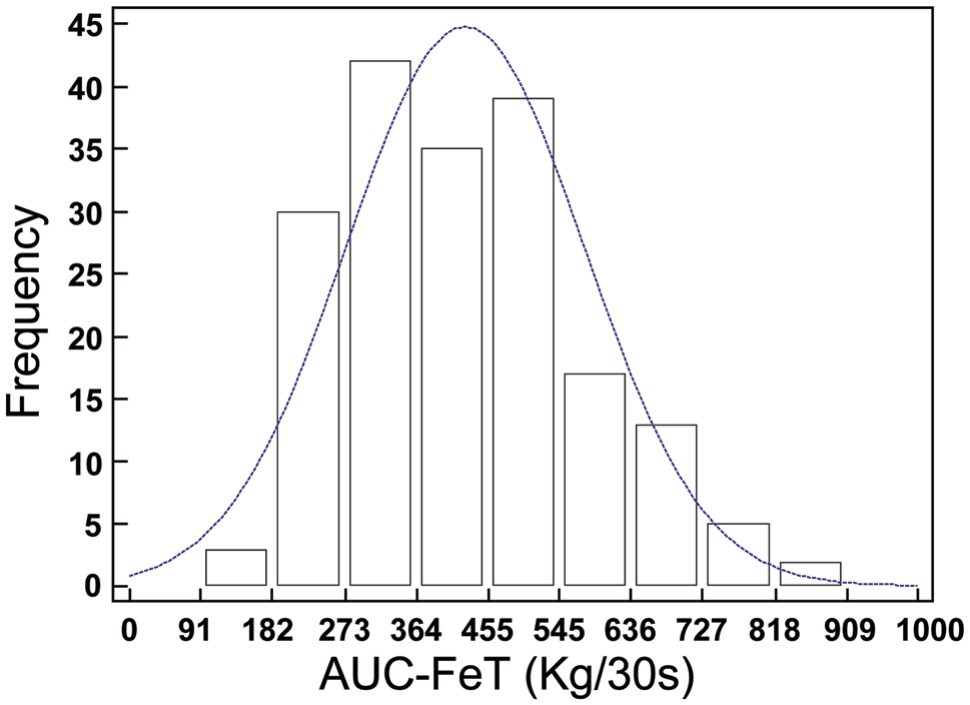
Distribution of the area under the force–time curve (AUC-FeT) scores

Figure 1 shows the estimation of central tendency and score distribution for the AUC-FeT scores, which resulted normal distributed.

Analyzing the relationships between the variables, Table 2 shows the correlations between handgrip strength findings and the other variables. The AUC-FeT was significantly correlated with age, SDAI, and US-CLARA (all with *p* < 0.0001), as well as with BMI (*p* = 0.0001). No correlations emerged with radiographic damage (SENS) and with comorbidities (mRDCI).

**Table 2:** Correlation coefficients (r) between hand grip strength features and other variables

	Peak grip force	Time to reach the plateau of the curve	BMI	Disease duration	Educational level	Age	mRDCI	SDAI	SENS	US-CLARA
AUC-FeT	0.668 < 0.0001	–0.312 < 0.0001	–0.279 0.0001	–0.157 0.031	–0.003 0.966	–0.321 < 0.0001	–0.040 0.591	–0.508 < 0.0001	–0.051 0.488	–0.501 < 0.0001
Peak grip force		–0.151 0.039	–0.227 0.0018	–0.036 0.630	0.110 0.136	–0.266 0.0002	–0.095 0.196	–0.186 0.010	0.032 0.668	–0.185 0.011
Time to reach the plateau of the curve			0.173 0.018	–0.012 0.868	–0.077 0.294	0.050 0.501	–0.016 0.831	0.513 < 0.0001	–0.090 0.222	0.537 < 0.0001
BMI				0.072 0.330	–0.005 0.940	0.094 0.202	–0.019 0.797	0.175 0.017	–0.045 0.537	0.194 0.007
Disease duration					0.033 0.653	0.101 0.171	0.041 0.581	0.182 0.013	0.771 < 0.0001	0.102 0.164
Educational level						0.050 0.496	–0.077 0.293	0.007 0.921	0.037 0.616	–0.079 0.281
Age							0.502 < 0.0001	0.085 0.250	0.054 0.465	0.120 0.104
mRDCI								–0.169 0.021	0.060 0.419	–0.099 0.177
SDAI									0.048 0.513	0.813 < 0.0001
SENS										0.030 0.687

*BMI* body mass index, *mRDCI* modified Rheumatic Disease Comorbidity Index, *SDAI* Simplified Disease Activity Index, *SENS* Simple Erosion Narrowing Score, *US-CLARA* Ultrasound-Clinical Arthritis Activity, *AUC-FeT* area under the force–time curve

**Fig. 2:**
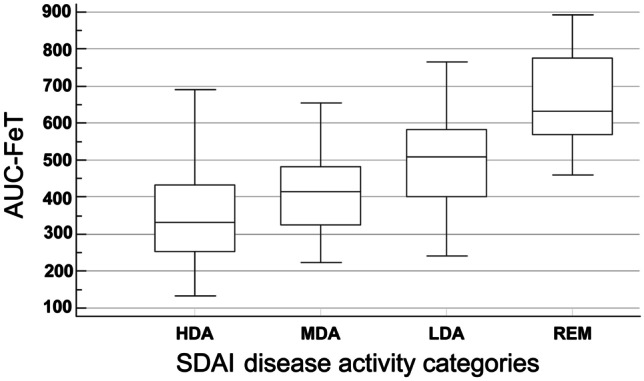
Box–Whisker plots showing the relationship between the AUC-FeT scores and the disease activity states evaluated with SDAI (one-way analysis of variance: ANOVA, F-ratio = 35.14, p < 0.001). The horizontal line in each box represents the median, and the box height represents the interquartile range

**Fig. 3:**
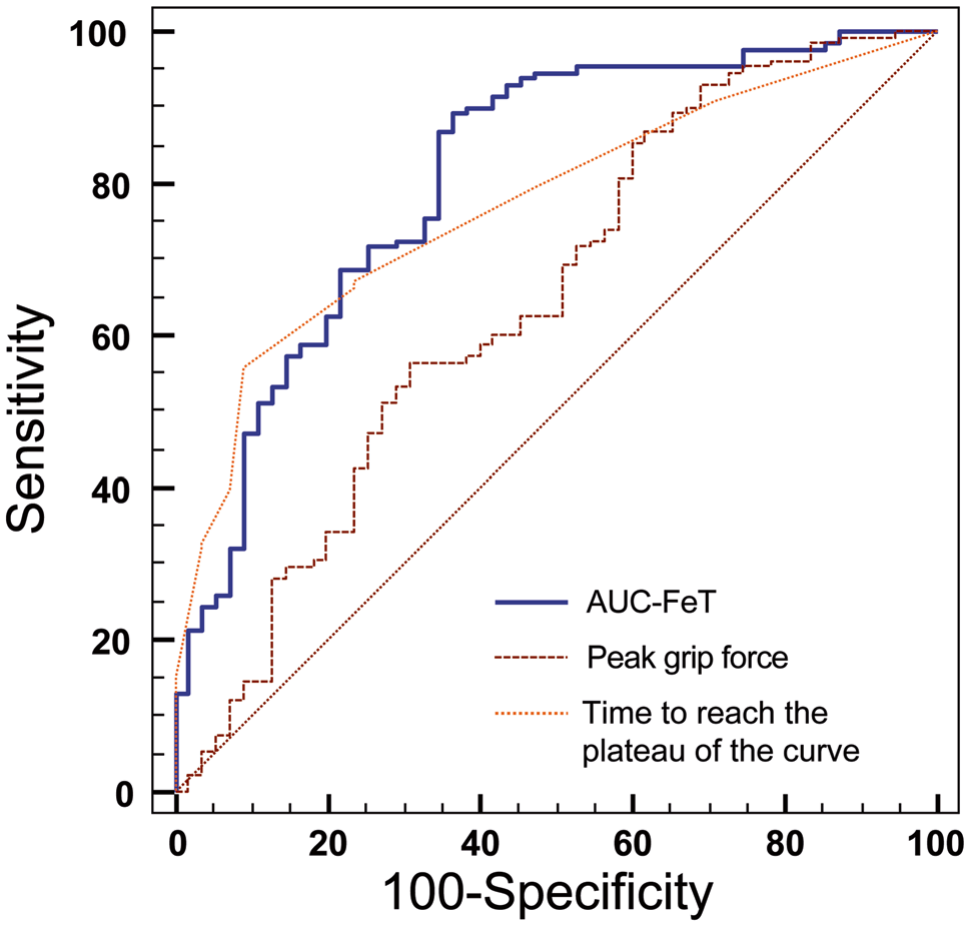
Receiver operating characteristic curve plots comparing the discriminative power of each of the three handgrip strength measures in the identification of patients with moderate/high disease activity

Categorizing patients according to SDAI categories, patients in HDA showed a significantly reduced AUC-FeT compared to patients in REM (ANOVA, F-ratio = 35.14, p < 0.001) (Fig. 2). The AUC-ROC analyses revealed that the best performance in identifying RA patients in MDA + HDA vs REM + LDA was provided by AUC-FeT [AUC-ROC = 0.810 (95% CI 0.746–0.864)], while peak grip force and time to reach the curve plateau were not statistically significant (Fig. 3).

Finally, the multivariate analysis reveale that the independent variables significantly associated with AUC-FeT were age (p = 0.0006), BMI (p = 0.012), sex (p = 0.004), SDAI (p = 0.047), and US-CLARA (p = 0.023) (Table 3).

**Table 3:** Variables associated with area under the force–time curve (AUC-FeT) in multivariate analysis

Independent variables	Coefficient	Standard error	t	p
(Constant)	997.2249			
Age	–2.5672	0.7370	–3.484	0.0006
Sex	–56.5544	19.7055	–2.870	0.0046
Education	–1.3249	2.3295	–0.569	0.5703
Disease duration	–2.5410	3.4591	–0.735	0.4636
BMI	–9.2652	3.6567	–2.534	0.0122
mRDCI	2.4787	6.3206	0.392	0.6954
SDAI	–2.4630	1.0631	–2.317	0.0217
US-CLARA	–14.1084	6.1664	–2.288	0.0233
SENS	0.02848	0.9633	0.0296	0.9764

*BMI* body mass index, *mRDCI* modified Rheumatic Disease Comorbidity Index, *SDAI* Simplified Disease Activity Index, *US-CLARA* Ultrasound-Clinical Arthritis Activity, *SENS* Simple Erosion Narrowing Score

## Discussion

HGs is one of the potential measures to capture muscular strength and is a simple and inexpensive risk-stratifying approach, having a prognostic value respect to all-causes mortality [24].

The objective of this study was to study the association between HGs features, estimated with an innovative cylindrical-shaped grip device, and demographic, anthropometric and clinical variables clinical variables in RA patients.

In previous studies, HGs has been found to be related with disease activity scores and disability [6]. The results of our study confirm previous findings, namely that reduced HGs correlates with more severe disease activity, but also with parameters such as age and BMI.

The main innovation introduced in this study is the way in which HGs was assessed. Typically, a HGs test simply assesses only the maximum grip strength. Conventional dynamometers such as the Jamar or Takei assess only unidirectional isometric force. In the presence of potentially very complex clinical manifestations such as rheumatoid hand, a unidirectional assessment may not fully reflect the strength and skill required to handle a cylindrical object. It has been demonstrated that the force required to handle a cylindrical object is greater than that used on traditional dynamometers, just as a cylindrical instrument more reliably assesses the force applied to the distal portion of the fingers [25]. Moreover, the cylindrical-shaped grip device used in this study, allowed other misurations such as the speed with which strength is generated, the ability to sustain strength, and the variability of handgrip strength. These parameters are overlooked when measuring only the peak force. The importance of using a cylindrical-shaped grip device has already been demonstrated by our research group in the context of fibromyalgia [26].

Beyond the tool used, an immediate difference emerged between men and women. Males patients have higher mean HGs than females, just as they do in the general population [24]. The impact of sex on muscular mass, maximum strength, and consequently maximal physiological reserve differs significantly [27]. Maximum HGs in healthy women has been observed to be 52–80% reduced of those in men [28]. The AUC-FeT values of HGs in this investigation were 25% lower in women than in men. As a result, women with RA may be more susceptible to functional deficits because to their weaker muscles.

The progressive loss of muscle mass and strength is one the most ineluctable anatomical alteration that happens with age [29–32], and our study confirmed that age is the strongest predictor of a reduced AUC-FeT.

Obesity was found to be inversely related with physical activity in previous cross-sectional studies, among a variety of genetic, physiological, behavioral, and environmental factors influencing physical activity in RA [33]. Researchers found that baseline BMI strongly predicted later self-reported physical activity patterns in Danish adult populations in two earlier studies [34, 35]. A more recent study showed long-term relationships between many adiposity indicators and moderate-to-vigorous physical activity [36]. Inflammation in RA promotes the loss of metabolically active tissue, and fat mass tends to rise. This syndrome, also known as "rheumatoid cachexia" or "sarcopenic obesity," can worsen the disease by causing lethargy, weakness, and decreased functional status [37].

Regarding disease activity, the HGs variables were strongly associated to the composite and multimodal disease activity indices. HGs, pinch measurements, and clinical and laboratory activity indices were all significantly associated to hand impairment [6, 38, 39]. According to our findings, incorporating US-imaging into a multimodal disease activity measure could improve the accuracy of such systems and have major consequences for patient classification [14].

Controversial is the relationship between HGs and radiological damage, and the findings of studies examining the direct link between radiographic deterioration and functional impairment remain equivocal. In our analysis, the radiological damage did not correlate with HGs, which is consistent with previous findings [40, 41]. Another study demonstrated that articular damage is inversely related to grip strength as a handicap marker in RA [42].

One of our study's major flaws is the cross-sectional design, thus any possible prognostic importance of HGs in RA patients has yet to be discovered. Furthermore, the research did not allowed to verify if the HGs responds to physical and/or medical treatment. The sensitivity of the HGs test in detecting changes in symptomatology following a therapeutic intervention should be evaluated in future investigations. Another limitation may be represented by the fact that HGs was evaluated only through the cylindrical-shaped device, without performing a comparison of the innovative device with traditional dynamometers. Finally, it can be criticized that the scoring of radiological damage has been performed through the SENS, a simplified method of a more articulated scoring system such as the SHS. However, SENS has proven to be a comparable tool to SHS, reliable and timesaving [15, 43].

In conclusion, our findings revealed that HGs was predicted by demographic (gender and age), anthropometric (BMI), and disease activity indices (SDAI and US-CLARA) in RA patients. Radiological damage was not a predictor of a reduced HGs. Assessing HGs as a separate variable, using a cylindrical-shaped device, could aid clinicians in better understanding how RA impairs function.

## Abbreviations

HDA: High disease activity
MDA: Moderate disease activity
LDA: Low disease activity
REM: Remission
